# Develop and validate a machine learning model to predict the risk of persistent pain after percutaneous transforaminal endoscopic discectomy

**DOI:** 10.3389/fsurg.2025.1631651

**Published:** 2025-07-23

**Authors:** Jun Yuan, Jun Fu

**Affiliations:** ^1^ Department of Orthopedics, Wuhan Hospital of Traditional Chinese Medicine, Wuhan, Hubei, China; ^2^ Department of Pain, Hubei Maternal and Child Health Hospital Affiliated to Tongji Medical College, Huazhong University of Science and Technology, Wuhan, Hubei, China

**Keywords:** lumbar disc herniation, machine learning, percutaneous transforaminal endoscopic discectomy, persistent pain, risk prediction model

## Abstract

**Background:**

Persistent pain is a common complication following percutaneous transforaminal endoscopic discectomy (PTED) for lumbar disc herniation. Identifying associated risk factors and developing a predictive model are crucial for guiding clinical decisions. This study aims to utilize machine learning models to predict persistent pain, identify key influencing factors, and construct a risk model to assess the likelihood of persistent pain.

**Methods:**

We first compared baseline characteristics and pathological indicators between patients who developed persistent pain and those who did not after PTED. Significant factors were used as input features in four machine learning models: Logistic Regression (LR), Support Vector Machine (SVM), XGBoost, and Multilayer Perceptron (MLP). Each model was optimized through grid search and 10-fold cross-validation. Performance was evaluated using ROC curves, F1 score, accuracy, recall, and precision. Models with AUC values exceeding 0.9, specifically XGBoost and MLP, were selected for SHAP visualization and risk prediction model construction.

**Results:**

Among the four machine learning models, XGBoost and MLP achieved the best performance, with AUC values of 0.907 and 0.916, respectively. SHAP analysis identified a history of lumbar spine trauma and herniation calcification as key features positively influencing persistent pain risk. Elevated inflammatory markers (e.g., CRP, ESR, and WBC) and older age also significantly impacted predictions. Using the most important features from XGBoost and MLP, a risk prediction model was constructed and externally validated, achieving an AUC of 0.798, indicating good predictive accuracy.

**Conclusion:**

History of lumbar spine trauma, herniation calcification, and inflammatory markers are important predictors of persistent pain after PTED. The risk prediction model based on XGBoost and MLP shows high predictive accuracy and can serve as a valuable tool for clinical decision-making.

## 1 Introduction

Lumbar disc herniation is a leading cause of lower back and leg pain, significantly affecting patients' quality of life and imposing substantial burdens on both society and families (1–3). With advancements in minimally invasive techniques, percutaneous transforaminal endoscopic discectomy (PTED) has emerged as a key treatment for lumbar disc herniation (4–6). This approach minimizes surgical incisions and reduces damage to surrounding tissues, offering benefits such as faster recovery and fewer complications, which has led to its widespread adoption in clinical practice (7). However, despite the overall success of the procedure, some patients continue to experience persistent postoperative pain, which extends the recovery period and adversely impacts their quality of life (8). The mechanisms behind this persistent pain are not yet fully understood, and accurately identifying high-risk patients before surgery remains challenging, complicating clinical management.

In recent years, machine learning has become increasingly prominent in medical research and clinical applications (9). Through training and optimization with large datasets, machine learning models can identify hidden patterns within complex data, significantly improving the accuracy and stability of disease prediction, especially for complex phenomena such as postoperative persistent pain (10). In this study, we applied four machine learning models—Logistic Regression (LR), Support Vector Machine (SVM) (11), XGBoost (12), and Multilayer Perceptron (MLP) (13)—to explore the effects of various features on persistent pain following PTED. By comparing the performance of these models, we selected the optimal model and used SHAP (Shapley Additive Explanations) values to interpret the impact of each feature on the prediction outcome, clarifying the role of key risk factors in persistent pain.

Through this research, we aim to identify the key factors contributing to persistent pain following lumbar disc herniation surgery and develop an effective risk prediction model. This model is intended to support clinical decision-making by helping clinicians identify high-risk patients prior to surgery, tailor individualized rehabilitation plans, and ultimately improve postoperative quality of life. We hope that the findings from this study will not only deepen the understanding of persistent pain after PTED but also provide valuable insights for managing other types of postoperative pain.

## 2 Materials and methods

### 2.1 Patient selection

Patients with lumbar disc herniation who underwent percutaneous transforaminal endoscopic discectomy at our hospital were retrospectively collected between May 2021 and May 2023 for the training cohort, and between June 2023 and June 2024 for the validation set. The former served as the training set, while the latter was used as the external validation set. Inclusion criteria were as follows: (1) age between 18 and 75; (2) grade of spinal spondylolisthesis ≤ Grade I; (3) Pfirrmann Grading of Grade II or Grade III; (4) lumbar segments involved were L3–L4, L4–L5, or L5–S1. Patients were divided into a persistent pain group and a non-persistent pain group based on the presence of persistent pain postoperatively. Exclusion criteria included: (1) presence of other diseases affecting the structure or function of the lumbar spine; (2) undergoing other interventions during follow-up that may influence lumbar pain (such as spinal fusion surgery or nerve block therapy); (3) severe hepatic or renal dysfunction; (4) serious systemic diseases such as malignancies; (5) severe mental or psychological disorders (e.g., major depression or severe anxiety) or poor treatment compliance; and (6) missing key data. Persistent pain was defined as a VAS score ≥ 4 and SF-36 scores < 50 in the pain and physical function domains at the 1-month postoperative follow-up. In the training set of 450 cases, 54 experienced postoperative persistent pain.

### 2.2 Data collection

We collected baseline characteristics and pathological indicators for each patient, including age, gender, BMI, history of lumbar spine trauma, duration of disease (whether more than 6 months), herniation calcification, spondylolisthesis grade, Pfirrmann Grading (14), lumbar segments (L3–L4, L4–L5, L5–S1), sagittal range of motion (sROM), and inflammatory indicators [C-Reactive Protein [CRP], Erythrocyte Sedimentation Rate [ESR], and White Blood Cell Count [WBC]]. These data were obtained from patient medical records and preoperative imaging results.

### 2.3 Machine learning model construction

To predict the occurrence of persistent postoperative pain, we selected four commonly used machine learning models for analysis: Logistic Regression (LR), Support Vector Machine (SVM), XGBoost, and Multilayer Perceptron (MLP). All input features were normalized before training to ensure consistency across different feature scales during model training. The DeLong test was used to perform pairwise comparisons of AUC values between models, with a *P*-value < 0.05 indicating a statistically significant difference in AUC.

### 2.4 Model tuning and validation

To optimize model performance, we employed grid search combined with 10-fold cross-validation to fine-tune the parameters of each model. In this process, the dataset was split into 10 subsets, with 9 subsets used for training and 1 subset for validation in each iteration. After completing all 10 iterations, the average performance metrics were calculated. The evaluation metrics included the area under the ROC curve (AUC), F1 Score, Accuracy, Recall, and Precision.

### 2.5 SHAP value analysis

To understand the prediction mechanism of the machine learning models, we used SHAP (Shapley Additive Explanations) values to explain the contribution of each feature to the prediction results (15). SHAP values quantify both the positive and negative impacts, as well as the importance of each feature on the prediction outcome, by assessing each feature's influence on the model output across different scenarios. We used SHAP values to analyze the direction and magnitude of each feature's effect on predicting persistent pain.

### 2.6 Risk model construction and validation

Based on the most important features identified through SHAP analysis, we constructed a simplified risk model to predict the risk of persistent postoperative pain. The primary formula was:

$$\mathrm{Risk}\;\mathrm{Index}=\mathrm{SHAP}*\mathrm{Featur}e_{\left[1\right]}+\mathrm{SHAP}*\mathrm{Featur}e_{\left[2\right]}+\mathrm{SHAP}*\mathrm{Featur}e_{\left[3\right]}$$

The SHAP values represent the average SHAP values from our chosen machine learning model. The top three important features were selected as input variables, and the model performance was validated on an external validation set. Model performance was evaluated using the AUC to assess its predictive capability.

### 2.7 Statistical analysis

All statistical analyses were conducted using R software, primarily utilizing the caret (16), iml, and shapviz packages. A *P*-value less than 0.05 was considered statistically significant. Continuous variables were presented as median (range) and compared using the Mann–Whitney *U*-test, while categorical variables were expressed as frequency (percentage) and analyzed with the Chi-square test or Fisher's exact test, as appropriate.

## 3 Results

### 3.1 Differences in baseline characteristics and pathological indicators between patients with and without persistent pain

The results showed that patients with persistent pain were older, had a higher proportion of lumbar spine trauma history, and a significantly higher percentage of disease duration exceeding 6 months. These patients also had higher rates of herniation calcification, mild spondylolisthesis (Grade I), and Pfirrmann Grade III, with a significant increase in L4-L5 segment involvement. Additionally, levels of inflammatory markers, including C-reactive protein (CRP), erythrocyte sedimentation rate (ESR), and white blood cell count (WBC), were higher in patients with persistent pain compared to those without (Table 1). The training set (*n* = 316) and external validation set (*n* = 134) showed a balanced distribution across multiple demographic characteristics such as age and BMI, as well as clinical indicators including disease duration and lumbar spondylolisthesis. There were no significant statistical differences between the two cohorts, indicating good comparability (Supplementary Table S1).

**Table 1:** Differences in basic characteristics and pathological indicators between patients with persistent postoperative pain and those without persistent postoperative pain.

Variables	All patients (*n* = 450)	No persistent pain (*n* = 396)	Persistent pain (*n* = 54)	*P*-value
Age (years)	43 (20–66)	42 (20–66)	50 (24–65)	0.0051
BMI (kg/m²)	26.89 (17.02–34.98)	26.89 (17.02–34.94)	27.16 (18.09–34.98)	0.815
Gender				0.2160683
Male	308 (68.44%)	275 (69.44%)	33 (61.11%)	
Female	142 (31.56%)	121 (30.56%)	21 (38.89%)	
Smoking				0.2513807
Yes	76 (16.89%)	64 (16.16%)	12 (22.22%)	
No	374 (83.11%)	332 (83.84%)	42 (77.78%)	
Drinking				0.2702325
Yes	87 (19.33%)	80 (20.2%)	7 (12.96%)	
No	363 (80.67%)	316 (79.8%)	47 (87.04%)	
History of lumbar spine trauma				1.90E-41
Yes	54 (12%)	10 (2.53%)	44 (81.48%)	
No	396 (88%)	386 (97.47%)	10 (18.52%)	
Course of Disease				0.001176945
≤6 Months	153 (34%)	145 (36.62%)	8 (14.81%)	
>6 Months	297 (66%)	251 (63.38%)	46 (85.19%)	
Herniation calcification				9.13E-21
Yes	57 (12.67%)	24 (6.06%)	33 (61.11%)	
No	393 (87.33%)	372 (93.94%)	21 (38.89%)	
Lumbar Spondylolisthesis				6.64E-18
Grade I	129 (28.67%)	85 (21.46%)	44 (81.48%)	
No	321 (71.33%)	311 (78.54%)	10 (18.52%)	
Spinal Canal Morphology				0.09771195
Cloverleaf Shape	65 (14.44%)	53 (13.38%)	12 (22.22%)	
Non-Cloverleaf Shape	385 (85.56%)	343 (86.62%)	42 (77.78%)	
Facet joint degeneration				0.06434208
Yes	431 (95.78%)	382 (96.46%)	49 (90.74%)	
No	19 (4.22%)	14 (3.54%)	5 (9.26%)	
Pfirrmann Grading				0.03438494
Grade II	158 (35.11%)	146 (36.87%)	12 (22.22%)	
Grade III	292 (64.89%)	250 (63.13%)	42 (77.78%)	
Lumbar Segments				0.04992969
L3–L4	47 (10.44%)	46 (11.62%)	1 (1.85%)	
L4–L5	210 (46.67%)	180 (45.45%)	30 (55.56%)	
L5–S1	193 (42.89%)	170 (42.93%)	23 (42.59%)	
C-Reactive Protein (mg/L)	11.88 (5.62–18.31)	11.68 (5.62–18.31)	13.88 (6.47–18.27)	0.00227
Erythrocyte Sedimentation Rate (mm/h)	19.07 (12.62–25.40)	18.90 (12.62–25.40)	20.60 (12.93–25.27)	0.00213
White Blood Cell Count (10^−9^/L)	7.10 (4.92–9.60)	6.99 (4.92–9.53)	8.13 (5.01–9.60)	0.000538
Sagittal Range of Motion (sROM)				0.4703628
<30°	246 (54.67%)	219 (55.3%)	27 (50%)	
≥30°	204 (45.33%)	177 (44.7%)	27 (50%)	

### 3.2 Selection of machine learning models

**Figure 1:**
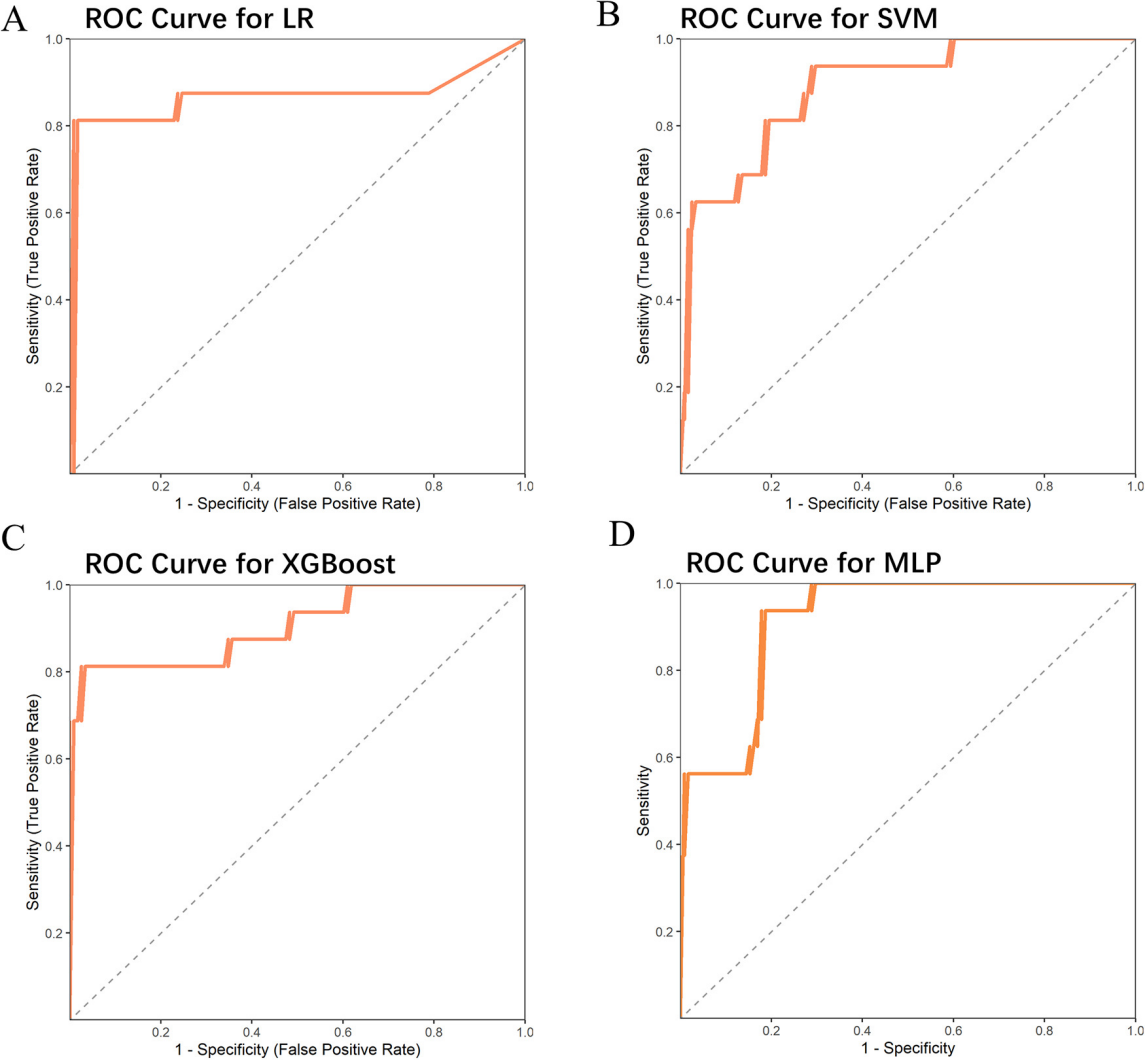
Four machine learning models’ ROC curves on the external validation set.

These significant factors were then input as independent variables into four machine learning models. The results showed that the ROC values for these four models were 0.867, 0.888, 0.907, and 0.916, respectively (Table 2) (Figure 1). Among them, the AUC values of the XGBoost and MLP models were both above 0.9, and these models also performed excellently in terms of F1 Score, Accuracy, Recall, and Precision. The DeLong test showed that the AUC values of the XGBoost and MLP models were significantly higher than those of the LR and SVM models (*P* < 0.05). Therefore, XGBoost and MLP were selected for visualization analysis (Table 3).

**Table 2:** ROC curve parameters of four machine learning models and risk indicators in the test Set and external validation Set.

Model	AUC	AUC_CI_Lower	AUC_CI_Upper	Best_Threshold	youden	Sensitivity	Specificity
LR	0.867	0.717	1.000	0.525	0.804	0.813	0.992
SVM	0.888	0.803	0.973	0.005	0.649	0.938	0.712
XGBoost	0.907	0.808	1.000	0.426	0.787	0.813	0.975
MLP	0.916	0.800	1.000	0.713	0.867	0.875	0.992
Risk Index	0.798	0.685	0.912	31.109	0.504	0.750	0.754

**Table 3:** Comparison of performance evaluation metrics for four machine learning models.

Model	F1 Score	Accuracy	Recall	Precision
LR	86.67	97.01	81.25	92.86
SVM	62.50	91.04	62.50	62.50
XGBoost	75.86	94.78	68.75	84.62
MLP	82.35	95.52	87.50	77.78

### 3.3 SHAP value explanation of model results

**Figure 2:**
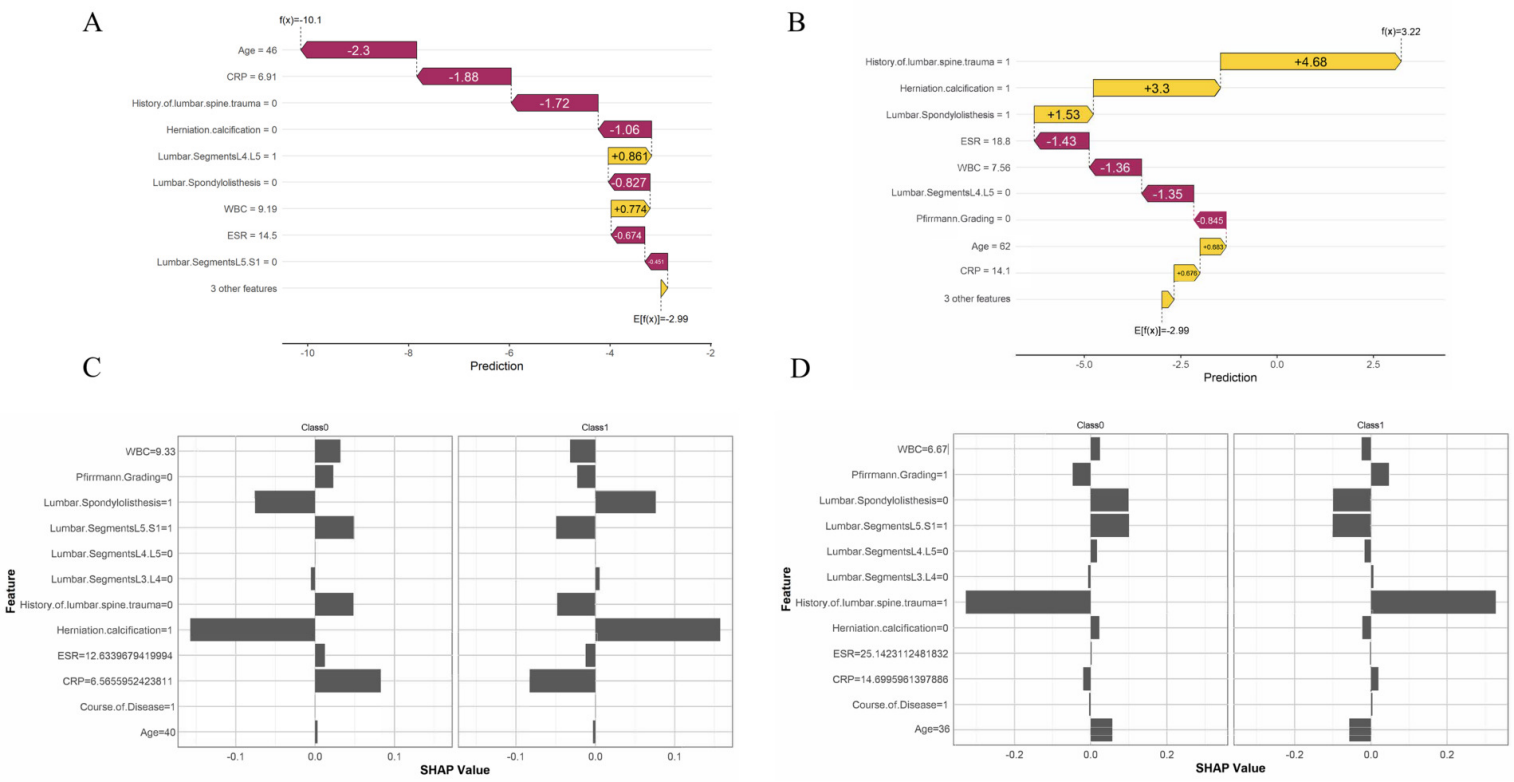
Model interpretation based on SHAP values. **(A)** A sample predicted by the XGBoost model not to experience persistent pain. **(B)** A sample predicted by the XGBoost model to experience persistent pain. **(C)** A sample predicted by the MLP model not to experience persistent pain. **(D)** A sample predicted by the MLP model to experience persistent pain.

For each machine learning model, a sample with persistent pain and a sample without persistent pain were randomly selected for analysis. The *x*-axis represents the direction of the change in the prediction value, with movement to the left indicating a negative influence and movement to the right indicating a positive influence. In Figure 2A, an age of 46 and CRP (C-Reactive Protein) level of 6.91 had a strong negative impact on the likelihood of persistent pain (−2.3 and −1.88, respectively), while certain lumbar segments and joint degeneration had a positive impact. The final expected prediction value was −2.99, indicating that the model predicts this patient is more likely not to experience persistent pain. In Figure 2B, a history of lumbar spine trauma (value of 1) and herniation calcification (value of 1) had a large positive impact on the prediction, significantly increasing the likelihood of persistent pain, with contributions of +4.68 and +3.3, respectively. Additionally, higher ESR (18.8) and WBC (7.56) levels had a negative impact on the prediction, reducing the risk of persistent pain.

In the MLP visualization results, in Figure 2C, for patients without persistent pain, herniation calcification and no history of lumbar spine trauma had a strong negative impact on the prediction. For patients with persistent pain, lumbar segment L5.S1 and herniation calcification had a significant positive impact on the prediction. In Figure 2D, a history of lumbar spine trauma had a strong positive impact on the prediction of persistent pain, while it showed a negative impact on the prediction of non-persistent pain, indicating that patients with a history of lumbar spine trauma are more likely to be predicted to experience persistent pain. Additionally, younger age also played a negative role in predicting the absence of persistent pain.

**Figure 3:**
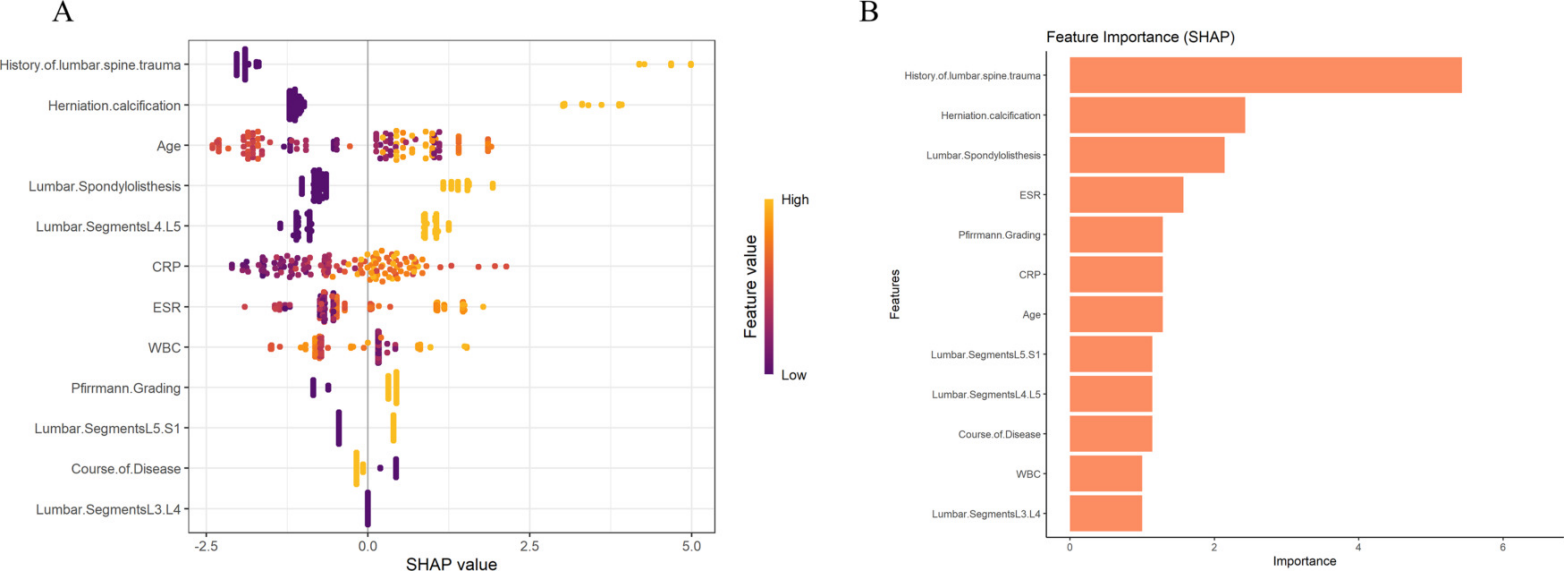
Visualization of feature importance and contribution to persistent pain risk based on **(A)** XGBoost and **(B)** MLP models.

The feature value analysis for all samples in Figure 3A shows that the *x*-axis represents SHAP values, indicating the direction and magnitude of each feature's impact on the prediction. The color gradient from purple to yellow represents the change in feature values from low to high. In the figure, a history of lumbar spine trauma (yellow for high values) and older age have a significant positive impact on the risk of persistent pain, indicating that higher values for these features increase the risk of persistent pain. High values for inflammatory indicators (e.g., CRP, ESR, and WBC) also have varying degrees of impact on the prediction. The MLP model results indicate that a history of lumbar spine trauma and herniation calcification are the most important features affecting the prediction outcome, contributing the most to the prediction of persistent pain, followed by spondylolisthesis, ESR, Pfirrmann Grading, and CRP (Figure 3B). We summarized the SHAP visualization results of the XGBoost and MLP models in Supplementary Table S2 to enhance clarity and interpretability. We also conducted visualization analysis in the external validation cohort, and the results showed that the feature rankings in the external validation set were similar to those in the training set, especially the top three most important features remained consistent. This further supports the stability and generalizability of the model (Supplementary Figure S1).

### 3.4 Construction and validation of the risk model

**Figure 4:**
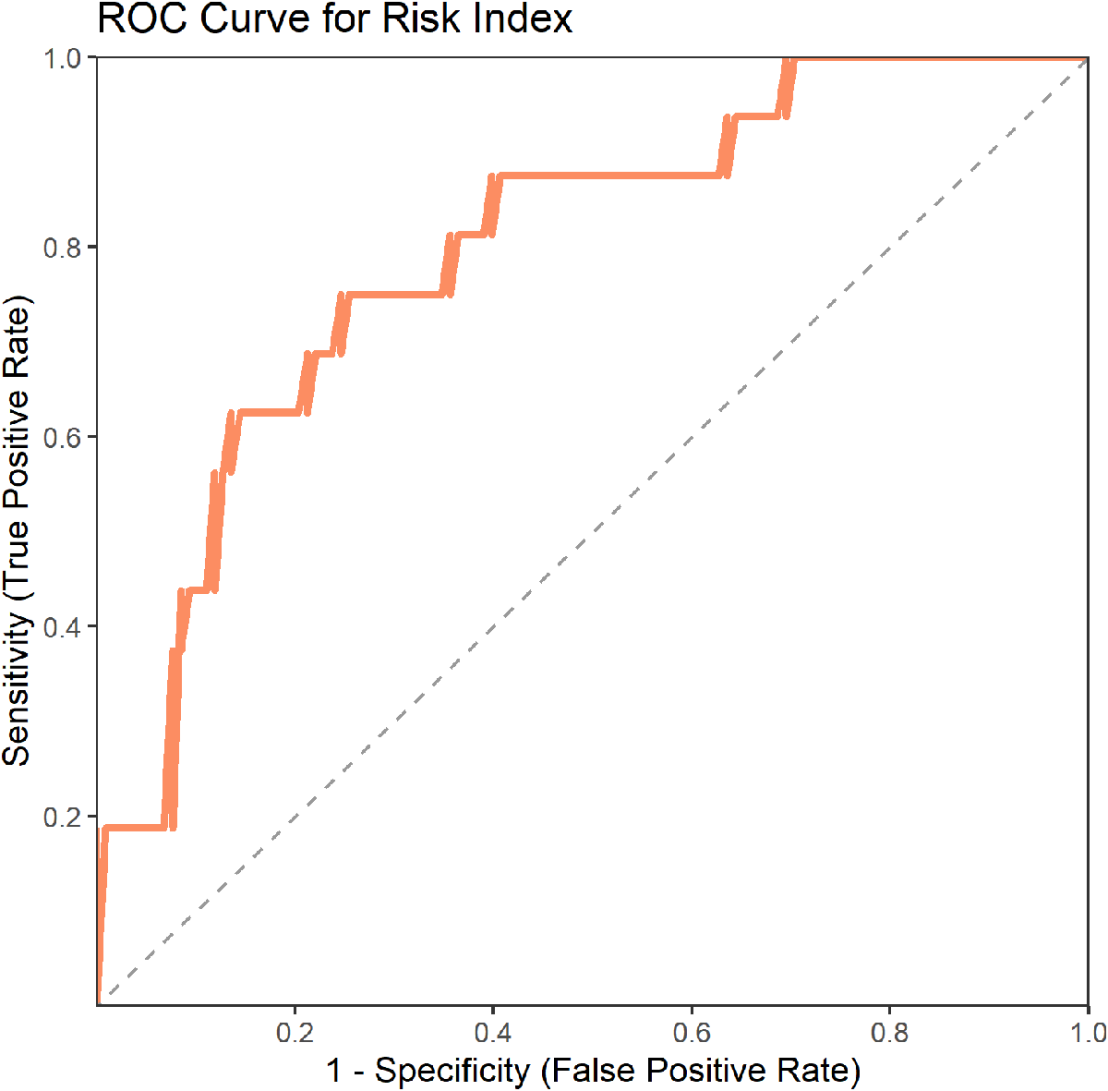
ROC curve for risk Index in external validation set.

We constructed a risk model to predict the likelihood of persistent pain based on the top three important features from these two machine learning models, and validated it in an external validation set. The results showed an AUC value of 0.798, indicating that the model has good predictive capability (Figure 4) (Table 2).

## 4 Discussion

This study is the first to apply machine learning techniques to identify multiple key risk factors for persistent pain following PTED surgery and successfully develop a machine learning-based risk prediction model to estimate the likelihood of persistent pain in patients. The findings highlight that a history of lumbar spine trauma and herniation calcification were the most influential features in both the XGBoost and MLP models, exhibiting significant positive effects on the risk prediction for persistent pain. These results suggest that anatomical changes and prior injury history may play crucial roles in the persistence of postoperative pain, while age and inflammatory status also appear to be important predictors.

We employed four machine learning models—Logistic Regression, Support Vector Machine, XGBoost, and Multilayer Perceptron—to compare their performance in predicting postoperative persistent pain and to identify the optimal model. These models captured the multi-level relationships between features and pain risk, with nonlinear models such as XGBoost and MLP demonstrating particular strength in uncovering complex feature interactions. Moreover, utilizing multiple models enhanced the stability and robustness of the predictions, minimizing potential biases inherent to any single model. Through SHAP value analysis of feature importance, this study offers clinicians valuable data-driven insights into the key factors influencing pain risk, thereby supporting more personalized treatment decisions.

Our study found a significant association between a history of lumbar spine trauma and the occurrence of persistent postoperative pain. Previous research has suggested that prior lumbar injuries may lead to structural and functional alterations in local nerves, muscles, and soft tissues, resulting in a sustained state of hypersensitivity (17). This heightened sensitivity makes these areas more susceptible to surgical stimulation and may lead to more pronounced postoperative pain responses (18, 19). Additionally, compensatory movement patterns and muscular imbalances developed after trauma may persist even after apparent recovery, potentially affecting spinal biomechanics and interfering with postoperative rehabilitation, thereby exacerbating the pain experience. Psychological studies have also indicated that individuals with a history of trauma are more prone to anxiety, depression, and other negative emotional states, which are closely linked to the development of postoperative pain (20, 21). These psychological factors may amplify pain perception through central sensitization mechanisms. Therefore, lumbar trauma may contribute to persistent postoperative pain through both physiological and psychological pathways.

Herniation calcification has a significant impact on postoperative pain, possibly because calcification hardens the protruding disc, increasing its pressure and friction on surrounding tissues (such as nerve roots, ligaments, and muscles) (22). Calcified tissue is challenging to remove during surgery, increasing the risk of damage to surrounding nerves and tissues, thereby intensifying postoperative pain (23). Calcification complicates surgical manipulation, particularly in PTED, as the hardness of the calcified disc limits the maneuverability of surgical instruments, making it difficult for surgeons to completely remove the herniated portion. Residual calcified tissue may continue to exert nerve compression postoperatively, resulting in persistent pain. Moreover, calcified disc tissue can provoke a more intense inflammatory response, which may delay tissue healing and contribute to chronic inflammation. This inflammation activates pain transmission pathways, heightening the patient's pain perception and prolonging the duration of postoperative pain. Elevated levels of CRP and ESR reflect a low-grade chronic inflammation within the patient's body (24). Intraoperative stimuli may promote the release of inflammatory mediators, thereby exacerbating tissue damage, inducing postoperative neural sensitization, and leading to postoperative edema, exudation, or adhesions, which cause persistent postoperative pain. Moreover, at higher CRP and ESR levels, the ability of soft tissue repair after surgery is reduced, making scar tissue formation more likely and aggravating postoperative discomfort.

The AUC value of our risk model was 0.798, indicating that this simplified model has good predictive ability and can be used as a quick assessment tool in clinical settings. In the future, it could be implemented as a mobile application, a risk prediction calculator, or integrated into electronic health record (EHR) systems, allowing clinicians to rapidly assess the risk of persistent postoperative pain before surgery. By identifying high-risk patients in advance, clinicians can optimize preoperative preparation and adjust treatment strategies accordingly, thereby improving surgical safety and patient outcomes.

However, this study has some limitations. The sample size is limited, which may restrict the model's generalizability. Future studies could expand the sample size to improve the model's stability and applicability across different populations. Persistent pain is a subjective experience, and there is currently a lack of a unified and widely accepted standard for its definition. Therefore, the generalizability of our study may be limited. Future research could incorporate more objective indicators to improve the assessment system for persistent pain and enhance the general applicability and clinical relevance of the findings.

## 5 Conclusion

This study identified several factors—including age, history of lumbar spine trauma, disease duration, and herniation calcification—that significantly increase the risk of persistent pain after PTED. The XGBoost and MLP models, built using these key factors, demonstrated excellent performance in predicting postoperative pain while offering strong interpretability. SHAP analysis highlighted lumbar spine trauma history and herniation calcification as the most influential predictors. The resulting risk prediction model aids in the preoperative identification of high-risk patients and supports the development of personalized intervention strategies.

## Data Availability

The original contributions presented in the study are included in the article/Supplementary Material, further inquiries can be directed to the corresponding author.

## Supplementary material

Supplementary Figure S1 Visualization of feature importance and contribution for persistent pain risk in the external validation cohort based on **(A)** XGBoost and **(B)** MLP models.
