# Supplementary material for: Develop and validate a machine learning model to predict the risk of persistent pain after percutaneous transforaminal endoscopic discectomy
Source: Front Surg. 2025 Jul 23;12:1631651. doi: 10.3389/fsurg.2025.1631651 (PMC12325245; doi:10.3389/fsurg.2025.1631651)
Supplement: Supplementary Figure S1 — Visualization of feature importance and contribution for persistent pain risk in the external validation cohort based on (A) XGBoost and (B) MLP models. [file Table1.docx]

| **Supplement Table 1. Comparison of Baseline Characteristics and Clinical Indicators between the Training and Validation Cohorts** | | | | |
| --- | --- | --- | --- | --- |
| **Variables** | **All Patients (n=450)** | **Training Set (n=316)** | **External Validation set (n=134)** | **P-value** |
| **Age (years)** | 43 (20-66) | 43 (20-66) | 41 (20-66) | 0.249 |
| **BMI (kg/m²)** | 26.89 (17.02-34.98) | 26.95 (17.03-34.98) | 25.82 (17.02-34.62) | 0.213 |
| **Gender** |  |  |  | 0.5368066 |
| Male | 308 (68.44%) | 213 (67.41%) | 95 (70.9%) |  |
| Female | 142 (31.56%) | 103 (32.59%) | 39 (29.1%) |  |
| **Smoking** |  |  |  | 0.607086 |
| Yes | 76 (16.89%) | 51 (16.14%) | 25 (18.66%) |  |
| No | 374 (83.11%) | 265 (83.86%) | 109 (81.34%) |  |
| **Drinking** |  |  |  | 0.6774638 |
| Yes | 87 (19.33%) | 59 (18.67%) | 28 (20.9%) |  |
| No | 363 (80.67%) | 257 (81.33%) | 106 (79.1%) |  |
| **History of lumbar spine trauma** |  |  |  | 6.52E-01 |
| Yes | 54 (12%) | 36 (11.39%) | 18 (13.43%) |  |
| No | 396 (88%) | 280 (88.61%) | 116 (86.57%) |  |
| **Course of Disease** |  |  |  | 0.5223007 |
| <=6 Months | 153 (34%) | 104 (32.91%) | 49 (36.57%) |  |
| >6 Months | 297 (66%) | 212 (67.09%) | 85 (63.43%) |  |
| **Herniation calcification** |  |  |  | 6.36E-01 |
| Yes | 57 (12.67%) | 38 (12.03%) | 19 (14.18%) |  |
| No | 393 (87.33%) | 278 (87.97%) | 115 (85.82%) |  |
| **Lumbar Spondylolisthesis** |  |  |  | 2.63E-01 |
| Grade I | 129 (28.67%) | 96 (30.38%) | 33 (24.63%) |  |
| No | 321 (71.33%) | 220 (69.62%) | 101 (75.37%) |  |
| **Spinal Canal Morphology** |  |  |  | 0.2582084 |
| Cloverleaf Shape | 65 (14.44%) | 50 (15.82%) | 15 (11.19%) |  |
| Non-Cloverleaf Shape | 385 (85.56%) | 266 (84.18%) | 119 (88.81%) |  |
| **Facet joint degeneration** |  |  |  | 0.1451109 |
| Yes | 431 (95.78%) | 306 (96.84%) | 125 (93.28%) |  |
| No | 19 (4.22%) | 10 (3.16%) | 9 (6.72%) |  |
| **Pfirrmann Grading** |  |  |  | 0.7379866 |
| Grade II | 158 (35.11%) | 113 (35.76%) | 45 (33.58%) |  |
| Grade III | 292 (64.89%) | 203 (64.24%) | 89 (66.42%) |  |
| **Lumbar Segments** |  |  |  | 0.613781 |
| L3-L4 | 47 (10.44%) | 33 (10.44%) | 14 (10.45%) |  |
| L4-L5 | 210 (46.67%) | 152 (48.1%) | 58 (43.28%) |  |
| L5-S1 | 193 (42.89%) | 131 (41.46%) | 62 (46.27%) |  |
| **C-Reactive Protein (mg/L)** | 11.88 (5.62-18.31) | 12.45 (5.62-18.31) | 11.15 (5.73-18.20) | 0.329 |
| **Erythrocyte Sedimentation Rate (mm/h)** | 19.07 (12.62-25.40) | 18.86 (12.62-25.40) | 19.28 (12.62-25.32) | 0.811 |
| **White Blood Cell Count (10^9/L)** | 7.10 (4.92-9.60) | 7.06 (4.96-9.60) | 7.13 (4.92-9.60) | 0.213 |
