# Supplementary material for: Develop and validate a machine learning model to predict the risk of persistent pain after percutaneous transforaminal endoscopic discectomy
Source: Front Surg. 2025 Jul 23;12:1631651. doi: 10.3389/fsurg.2025.1631651 (PMC12325245; doi:10.3389/fsurg.2025.1631651)
Supplement: Supplementary file 2 [file Table2.docx]

| **Supplement Table. 2 Important Features Identified by SHAP Analysis in XGBoost and MLP Models** | | |
| --- | --- | --- |
| **Variables** | **XGBoost** | **MLP** |
| History of lumbar spine trauma | 2.164 | 5.429 |
| Herniation calcification | 1.389 | 2.429 |
| Age | 1.155 | 1.286 |
| Lumbar Spondylolisthesis | 1.023 | 2.143 |
| Lumbar SegmentsL4-L5 | 0.997 | 1.143 |
| CRP | 0.816 | 1.286 |
| ESR | 0.813 | 1.571 |
| WBC | 0.553 | 1.000 |
| Pfirrmann Grading | 0.535 | 1.286 |
| Lumbar SegmentsL5-S1 | 0.427 | 1.143 |
| Course of Disease | 0.252 | 1.143 |
| Lumbar SegmentsL3-L4 | 0.000 | 1.000 |
