# Supplementary figures and images for: Develop and validate a machine learning model to predict the risk of persistent pain after percutaneous transforaminal endoscopic discectomy
Source: Front Surg. 2025 Jul 23;12:1631651. doi: 10.3389/fsurg.2025.1631651 (PMC12325245; doi:10.3389/fsurg.2025.1631651)

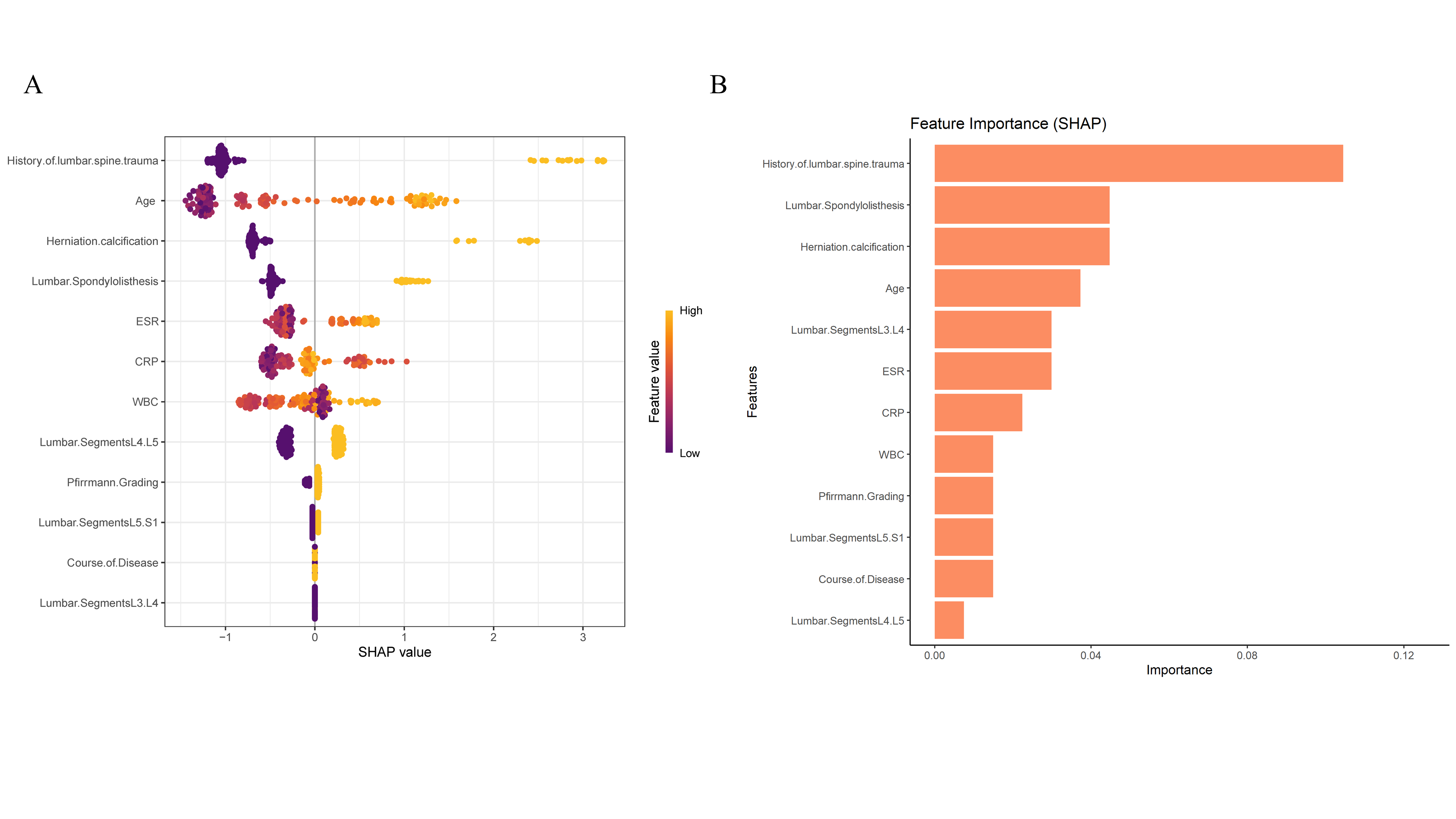

Supplement: Supplementary file 3 [file Image1.png]
